# Multimodal Imaging of a Chimney-Stenting Procedure Performed Simultaneously with a Transcatheter Aortic Valve Replacement (TAVR) in a Reanimated Human Heart including Post-Implant Analyses

**DOI:** 10.3390/jcdd9120413

**Published:** 2022-11-24

**Authors:** Michael A. Bielecki, Amanda N. DeVos, Francesco Bianchini, Paul A. Iaizzo

**Affiliations:** 1 Departments of Surgery and Biomedical Engineering, Institute for Engineering in Medicine, University of Minnesota, Minneapolis, MN 55455, USA; 2 Cardiology, Fondazione Policlinico Universitario Agostino Gemelli, 00168 Metropolitan City of Rome, Italy

**Keywords:** Evolut^™^ Pro+, transcatheter aortic valve replacement (TAVR), chimney, percutaneous coronary intervention (PCI), post-TAVR PCI, deployment, post-procedural imaging, micro-computed tomography (micro-CT), computational reconstructions

## Abstract

Transcatheter aortic valve replacement (TAVR) has become a popular treatment option for severe aortic stenosis for patients with a high risk for mortality with surgical aortic valve replacement (SAVR). Coronary artery occlusion (CAO) following the implantation of the device is a potential and sometimes devastating complication of this procedure, that provokes a sudden deterioration of hemodynamic status followed by cardiogenic shock and electrical instability. With patients that present a high risk for coronary obstruction, coronary protection with a chimney stenting technique is an effective strategy that can ensure coronary perfusion during TAVR in case of acute CAO. Utilizing Visible Heart^®^ methodologies, a human heart was reanimated. A chimney stenting technique was implemented simultaneously with the deployment of a Medtronic Evolut^™^ Pro+ valve (Medtronic PLC; Minneapolis, MN, USA). The entire procedure was recorded utilizing endoscopic cameras, fluoroscopy, optical coherence tomography, and echocardiography. In addition to these procedural visualizations, post-procedural micro-computed tomography (micro-CT) was conducted to provide post-implantation imaging with approximately 60-micron resolution. Utilizing these imaging modalities in a reanimated human heart allows for the unique opportunity to collect data for TAVR procedures in real human anatomies for the subsequent educational uses by the physicians treating aortic valvular disease and/or the designers of future TAVR technologies and procedures.

## 1. Introduction

Transcatheter aortic valve replacements (TAVR) are an alternative to surgical aortic valve replacement (SAVR) for patients with symptomatic severe aortic stenosis, in spite of the preoperative risk and considering the most recent guidelines and trial results [1]. Such procedures have recently surpassed all surgical aortic valve replacements (SAVR) in numbers, as the most common treatment for aortic stenosis (AS) or aortic insufficiency (AI) [1,2,3]. For instance, there were roughly 73,000 TAVRs compared to about 58,000 SAVR procedures conducted in the United States, in 2019 [3]. During a TAVR procedure, a prosthetic valve is delivered typically via a delivery catheter through the femoral artery and arterial vasculature until it reaches the aortic annulus. From here, the valve is deployed across the annulus, assuming the function of the aortic valve. The structure of the prosthesis displaces the diseased leaflets and improves systolic blood flow from the left ventricle. While the adoption of TAVR has rapidly increased since the early 2000s due in part to the clinical evidence proving that it is a safe and effective solution for AS or AI, there are still questions regarding some of the infrequent complications that are associated with TAVR procedures.

One potentially devastating complication that can occur with TAVR procedures are obstructions of the coronary arteries, due to either the native leaflets of the valve or the structure of the TAVR itself imposing obstructions. This type of intra-procedural complication can in turn provoke a sudden deterioration of hemodynamic status, followed by cardiogenic shock, electrical instability, and/or prevent further access to the coronaries for needed future procedures. In patients at high risk for coronary obstructions, coronary protection with the performance of chimney stenting is a technique that can aid to ensure coronary perfusion during the TAVR procedure in cases with the potential for acute coronary artery occlusion (CAO) [4]. Typically, before implementing a TAVR, pre-procedural imaging is collected to obtain insights into prosthesis sizing and for assessing the risks of TAVR, such as coronary occlusion. If the imaging shows that there are significant risks of coronary obstruction, prior to the valve deployment, a chimney technique can be implemented. In the chimney technique, a wire is commonly advanced in the coronary artery and a stent is placed distally before the valve prosthesis is deployed. The guide catheter is then retracted, and the stent is deployed into the coronary ostium simultaneously with the deployment of the valve prosthesis causing the stent to wrap around the outside of the TAVR, where the opening of the stent is then flared with the stent balloon, allowing for continued coronary perfusion. Typically, the TAVR and chimney techniques can take place within 90–120 min procedure times: depending on the prosthesis type and placement technique.

If a patient presents with a high risk for acute CAO, a SAVR may be the more appropriate treatment option. However, if the patient is considered unable to withstand the surgical approach, other procedural techniques, such as the chimney, should be considered. The chimney technique is described as a protective percutaneous coronary intervention (PCI) to prevent acute CAO. Note, the reported 30-day mortality rate of acute CAO ranges widely between 8% and 41% [5]. With this high mortality rate, protective and bailout procedures, such as the chimney technique, have been developed and used to reduce these numbers. A chimney technique may be preferable to other bailout techniques, as it can be implemented simultaneously with the TAVR, reducing the total procedural time.

The Visible Heart^®^ Laboratories have developed and utilized ongoing methodologies for in vitro testing of TAVR and other related procedures, including simultaneous chimney stenting. This preclinical research approach also allows for the unique, but rare, reanimation of human hearts; donated for research through a collaboration with LifeSource Inc (Minneapolis, MN, USA) [6,7]. As such, the aortic and adjacent anatomies can be directly visualized, in the heart’s native sinus rhythm, during such procedures [6,7]. These human hearts often present with unique disease states, such as AS and AI; those disease states caused them to be deemed not viable for transplant. Nevertheless, these gifted specimens allow for novel TAVR testing in real human specimens with varied anatomies that cannot be matched in other testing models.

A chimney stenting and TAVR procedures were conducted while employing the Visible Heart^®^ methodologies. Procedural imaging, including endoscopes, fluoroscopy, and echocardiography was utilized. Post-implant procedures, formalin perfusion fixation was performed, followed by post-procedural micro-CT imaging for this same specimen, so to generate 3D models of the procedures for analyses of resultant device placements or technique results. The effects of post-TAVR PCI are not well defined in the literature, and there remains a demand from physicians for better educational resources associated with these complex procedures. This work uses the Visible Heart^®^ imaging methodologies to display the specific features of a simultaneous chimney stenting/ TAVR technique in functional human anatomies, as means to better address these educational needs.

## 2. Materials and Methods

**Figure 1:**
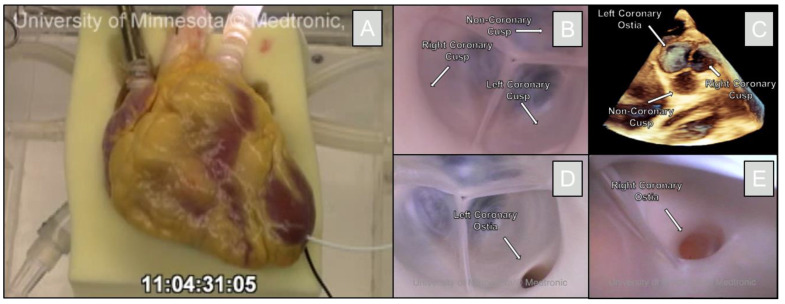
Reanimation and visualization of the aortic valve using Visible Heart^®^ methodologies. The human heart is shown on the Visible Heart^®^ apparatus (**A**). An endoscopic camera shows the aortic valve (**B**). A 3D-echo view shows the aortic valve and corresponding cusps (**C**). The endoscopic camera was able to visualize each of the left coronary ostium (**D**), and the right coronary ostium (**E**).

The organ donation came from a 27-year-old female who was declared brain dead, and her heart was deemed nonviable for transplantation. Informed consent was collected from the patient’s relatives, and the heart was recovered en-bloc using LifeSource protocols and donated to the University of Minnesota for research. The heart was prepared for reanimation in less than 8 h post-cross-clamp. This included the process of removing the lungs from the heart and cannulation of the great vessels. The newly isolated heart was attached to the Visible Heart^®^ apparatus for functional right and left-sided heart circulations [6,7]. A transparent Krebs-Henseleit buffer perfused the heart at a normal body temperature (roughly 37 °C). Dobutamine and nitroglycerin were injected into the solution prior to a 30-joule defibrillation shock. After the defibrillation, the heart was able to sustain function in a normal sinus rhythm for >5 h. Figure 1 shows the heart specimen during these procedures.

**Figure 2:**
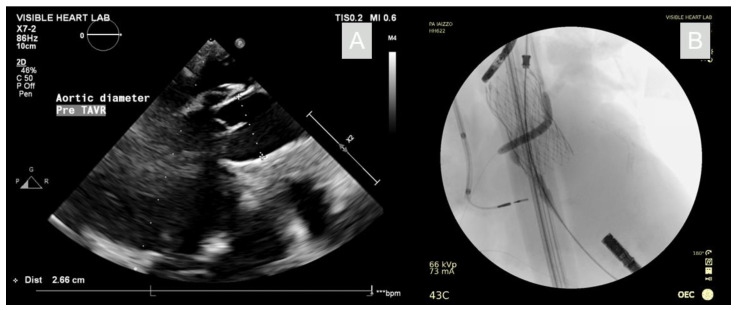
Echocardiography with aortic diameter measurement for prosthesis sizing (**A**). Fluoroscopy of the deployed TAVR and chimney stent with deployment balloon inflated (**B**).

Following the reanimation, it was shown that the anatomic position of the right coronary ostium lay very deep in the coronary cusp, proximal to the annulus. Therefore, if a TAVR procedure alone was conducted in this specimen, the right coronary vessel would likely have a high risk for occlusion. With this in mind, it was determined that, while the aortic valve did not pose indications for a TAVR procedure, we were presented with a unique opportunity to visualize a simultaneous chimney-stenting procedure in functional human anatomies. The aortic annulus was assessed to have a ~26 mm diameter when imaged apically using echocardiography, as seen in Figure 2. Hence, a corresponding 26 mm Evolut™ Pro+ valve was selected and loaded into an 18Fr EnVeo™ delivery system and advanced over a guidewire through the aorta and across the aortic valve. A pigtail catheter was positioned in the non-coronary cusp of the aortic valve prior to the valve deployment. Additionally, a guide wire was passed through the right coronary ostium via a 6F coronary guide catheter (Medtronic, MN, USA). An optical coherence tomography (OCT) scan was performed by advancing a Dragonfly Optis™ catheter (Abbott, IL, USA) over the guidewire and into the coronary artery. A pullback was performed such that an intravascular cross-sectional image was taken every 0.1 mm over 54 mm of a vessel. From the OCT images, the coronary diameter was determined and a 4 mm diameter, 22 mm length Onyx stent (Medtronic, MN, USA) was selected. The stent was advanced over the guidewire, then positioned so it would extend out of the coronary vessel and into the aorta.

## 3. Results

**Figure 3:**
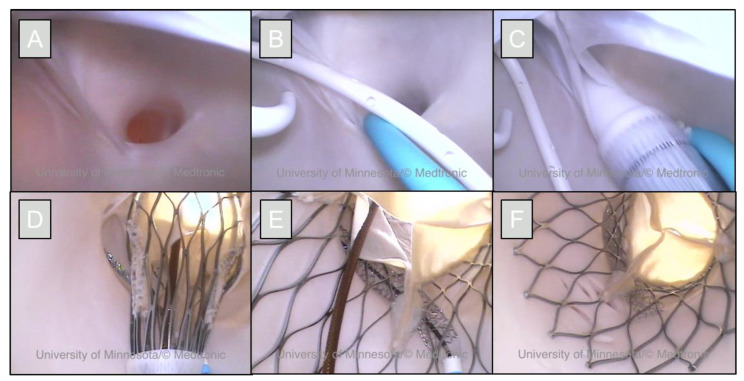
Endoscopic views of a chimney stenting procedure in a reanimated human heart. The right coronary ostium of this heart presented as very low in the right coronary cusp (**A**); making it a good candidate for performing a chimney technique. A pig-tail catheter is placed in the noncoronary cusp, and the stent was placed using a guide catheter in the right coronary ostium (**B**). The TAVR delivery catheter was placed across the aortic annulus (**C**). The prosthetic valve was partially deployed to ensure proper placement and the stent was exposed from its guide catheter (**D**). The TAVR and stents were then simultaneously deployed (**E**). The guide catheter and delivery catheters were subsequently removed (**F**).

**Figure 4:**
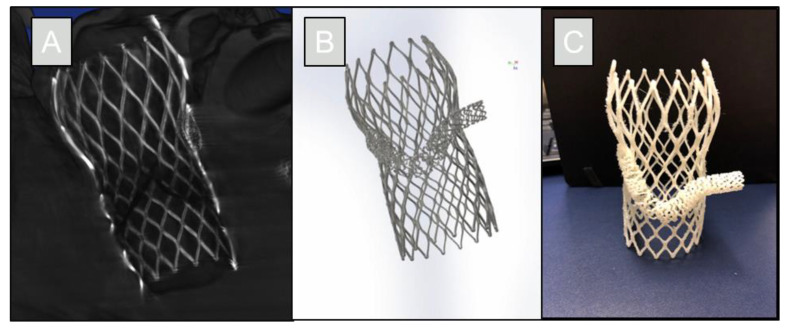
The reconstruction was made from a micro-CT scan with ~55-micron resolution (**A**). The reconstruction was segmented and converted into a computational model of the chimney technique (**B**). This model can then be used as an educational tool, including 3D printing of the resultant procedure (**C**).

The prosthesis was deployed while simultaneously inflating the stent, allowing for the stent to wrap around the outside of the valve without being deformed. The guide wires and delivery catheters were removed, and the prosthesis and chimney technique were visualized with endoscopic cameras to assess the results of the technique as shown in Figure 3. The heart was then removed from the apparatus and perfusion fixed using a 10% buffered formalin solution, while pressurized at roughly 50 mmHg through the great vessels [8]. This fixation process preserves the aortic root anatomy and the implanted valve in an approximate end-diastolic state. Following fixation, the heart was then scanned on an X3000 high-resolution micro-CT system (North Star Imaging, Rogers, MN, USA) and anatomies, the TAV and stent reconstructed with the corresponding efX-ct software, shown in Figure 4, for further analyses.

Following the procedure, direct visualizations showed that there was still access to the left coronary ostium even with the TAVR prosthesis. The stent was engaged into the coronary cusp and the vessel; allowing for coronary perfusion. The reconstruction was segmented using Mimics Materialise software (Materialise NV; Leuven, Belgium). The generated computational model of the chimney procedure was then printed using a uPrint S plus 3D printer (Stratasys, Ltd.; Eden Prairie, MN, USA) to create a physical model, shown in Figure 4.

## 4. Discussion

The anatomical presentation of low proximal positioning of the coronary ostium within the cusp provided a useful research opportunity to perform a chimney stenting procedure simultaneously with a TAV placement within a functional human heart. The stent allowed for flow access and perfusion of the coronary ostium following the prosthesis placement. Proper placement of the valve prosthesis and chimney stent was achieved using direct visualization through endoscopic, fluoroscopic, and echocardiographic imaging techniques as commonly implemented within the Visible Heart^®^ Laboratories.

The direct visualizations of the TAVR and chimney placements in functional human anatomy on the Visible Heart^®^ apparatus can be used in teaching modules for future physicians and designers of TAVR procedures; to show the effects of these techniques in educational footage. Video collected from the fluoroscopy and echocardiography can be correlated to the endoscopic footage to guide proper placement techniques using the more limited clinically available imaging. Furthermore, the post-implant analyses provide designers of TAVR and associated procedures with valuable information regarding how the frame of the device may affect coronary access and their positionings within the ascending aorta. It is planned that these methodologies will be used in other TAVR-PCI techniques such as the snorkel stenting technique, and with other valve-in-valve procedures to expand these educational resources.

## 5. Conclusions

Visualizations collected using Visible Heart^®^ methodologies highlight the importance of proper TAVR positioning as well as the proper chimney placement. The imaging methodologies and direct visualizations of these techniques allowed for both optimal prosthesis and stent deployments. The post-procedural micro-CT imaging allowed for the generation of computational reconstructions to develop unique educational models that correspond with real human anatomies. All described methodologies will be used for future performed TAVR and TAVR-PCI techniques and technologies. The pre-clinical methodologies employed here provide unique educational assets for the training of physicians and can provide critical insights into the development of TAVR and associated techniques.

## Data Availability

All video footage will be uploaded to the Visible Heart^®^ Laboratories’ Atlas of Human Cardiac Anatomy (http://www.vhlab.umn.edu/atlas/) (29 September 2022), a free access website cataloging the assessed and reanimated human heart anatomies donated to the laboratories.
